# Antimicrobial Peptides as Potential Antiviral Factors in Insect Antiviral Immune Response

**DOI:** 10.3389/fimmu.2020.02030

**Published:** 2020-09-02

**Authors:** Min Feng, Shigang Fei, Junming Xia, Vassiliki Labropoulou, Luc Swevers, Jingchen Sun

**Affiliations:** ^1^Guangdong Provincial Key Laboratory of Agro-Animal Genomics and Molecular Breeding, College of Animal Science, South China Agricultural University, Guangzhou, China; ^2^Insect Molecular Genetics and Biotechnology, Institute of Biosciences and Applications, National Centre for Scientific Research Demokritos, Athens, Greece

**Keywords:** antiviral peptides, antimicrobial peptides, insect, viruses, antiviral drugs

## Abstract

Antimicrobial peptides (AMPs) with antiviral activity (antiviral peptides: AVPs) have become a research hotspot and already show immense potential to become pharmaceutically available antiviral drugs. AVPs have exhibited huge potential in inhibiting viruses by targeting various stages of their life cycle. Insects are the most speciose group of animals that inhabit almost all ecosystems and habitats on the land and are a rich source of natural AMPs. However, insect AVP mining, functional research, and drug development are still in their infancy. This review aims to summarize the currently validated insect AVPs, explore potential new insect AVPs and to discuss their possible mechanism of synthesis and action, with a view to providing clues to unravel the mechanisms of insect antiviral immunity and to develop insect AVP-derived antiviral drugs.

## Introduction

The role that insects have played as models in innate immunity research is unquestionable. Since the 1990's, the fruit fly *Drosophila melanogaster* emerged as an important paradigm of genetic analysis of innate immunity. Outstanding pioneering achievements were awarded the Nobel Prize, which has since greatly stimulated interest in this field (1, 2). Studies in insects initially focused on resistance to bacteria and fungi, and later slowly expanded into antiviral immunity. However, besides the discovery that RNA interference (RNAi) is crucial in insect antiviral immunity, knowledge of other antiviral pathways and antiviral factors is very limited (3–7). In contrast, in mammals, a diverse series of antiviral immune responses including virus recognition, downstream cascade reactions, and production of effectors were gradually unveiled (8–10). In particular, hundreds of interferon-stimulated genes (ISGs), which exert numerous antiviral effector functions, have been identified in multiple vertebrate species (11–15). This raises the question whether antiviral host factors, similar to interferon-stimulated effectors in mammals, also exist in insects.

In insects, antimicrobial peptides (AMPs) are a group of immune proteins that mainly function against bacteria and fungi (16, 17). A considerable number of AMP genes have been identified in *Drosophila*, the honey bee *Apis cerana* and the silkworm *Bombyx mori* (18–20). However, two antiviral screening experiments failed to show that AMPs are a class of antiviral factors in *Drosophila* (21, 22). Intriguingly, other data in the literature have indicated that AMPs have antiviral function in *Drosophila* and *B. mori* (23, 24). On the other hand, it should be kept in mind that the interaction between host and virus is a complex process in which the immune response of the host is counteracted by the immune escape mechanisms of the virus. A recent study found that Kallithea virus (DNA virus of *D. melanogaster*) gp83 inhibits Toll signaling through the regulation of NF-κB transcription factors (25). The immunosuppression by Kallithea virus infection is also accompanied by the general down-regulation of AMP gene expression (25). Because the action of AMPs may be neutralized by the virus, simple tests cannot decide or exclude whether AMPs have antiviral activity. In fact, AMPs with antiviral activity (antiviral peptides: AVPs) have become a research hotspot and already show considerable potential to become pharmaceutically available antiviral drugs (26). AMPs and AVPs are usually derived from natural sources but they can be readily modified by adding non-natural amino acids or chemical groups to further enhance their stability and activity (27). Insects are an extremely successful and diverse group of animals that produce a wide range of AMPs which also could display potent antiviral activity. Accordingly, a review of insect antibacterial peptides with antiviral activity is considered timely to provide an assessment of the current knowledge as well as to stimulate efforts for the identification of additional insect-derived antiviral AMPs.

Herein, we will summarize the AMPs with antiviral activity reported in the database and literature and we will predict the antiviral activity of insect AMPs through AVP prediction software. This article aims to compile relevant information from insect AVPs as important components of insect antiviral innate immunity and to inspire the development of effective antiviral drugs.

## Databases and Websites of Insect AVPS

AVPs are considered as a subset of AMPs which act as the first line of defense in many organisms as an innate immune response to viral infection. Compared to a hot field such as the development of antiviral and antitumor drugs in human medicine, the concept of AVP has not appeared often in the field of insect research, although the idea appeared more than 10 years ago (28, 29). With increasing interest for natural AMPs as potential new drugs, many databases, such as APD (30), AVPdb (31) and ParaPep (32), have been developed to centralize information about AMPs. Among AMP databases, a few databases integrate the AMPs with antiviral activity such as APD (30), AVPdb (31), DRAMP 2.0 (33), and dbAMP (34). The information incorporated in DRAMP 2.0 and dbAMP is relatively new and complete. The advantage of AVPdb is that it summarizes AVPs according to various anti-virus mechanisms. In addition, software for AVP prediction has been developed, e.g., AVPpred (35), AntiVPP 1.0 (36), and Meta-iAVP (37). Based on a series of concepts relevant to insect AVP research, we have cataloged several user-friendly and recently released databases and websites that are suitable for insect AVP research (Table 1). The data of known AVPs and prediction methods in this article also come from these databases and websites.

**Table 1:** Databases and websites suitable for insect AVP research.

**Name**	**Websites**	**Function**	**References**
dbAMP	http://csb.cse.yzu.edu.tw/dbAMP/	Search for AVPs and insect-derived AMPs	(34)
DRAMP 2.0	http://dramp.cpu-bioinfor.org/	Search for insect AVPs	(33)
AVPdb	http://crdd.osdd.net/servers/avpdb/index.php	Antiviral mechanism of AVPs for reference	(31)
SignalP-5.0	http://www.cbs.dtu.dk/services/SignalP/	Prediction of AMPs signal peptide	(38)
Meta-iAVP	http://codes.bio/meta-iavp/	Prediction of AVPs	(37)

## Insect AMPS With Antiviral Activities: The Insect AVPS in Public Databases

**Figure 1:**
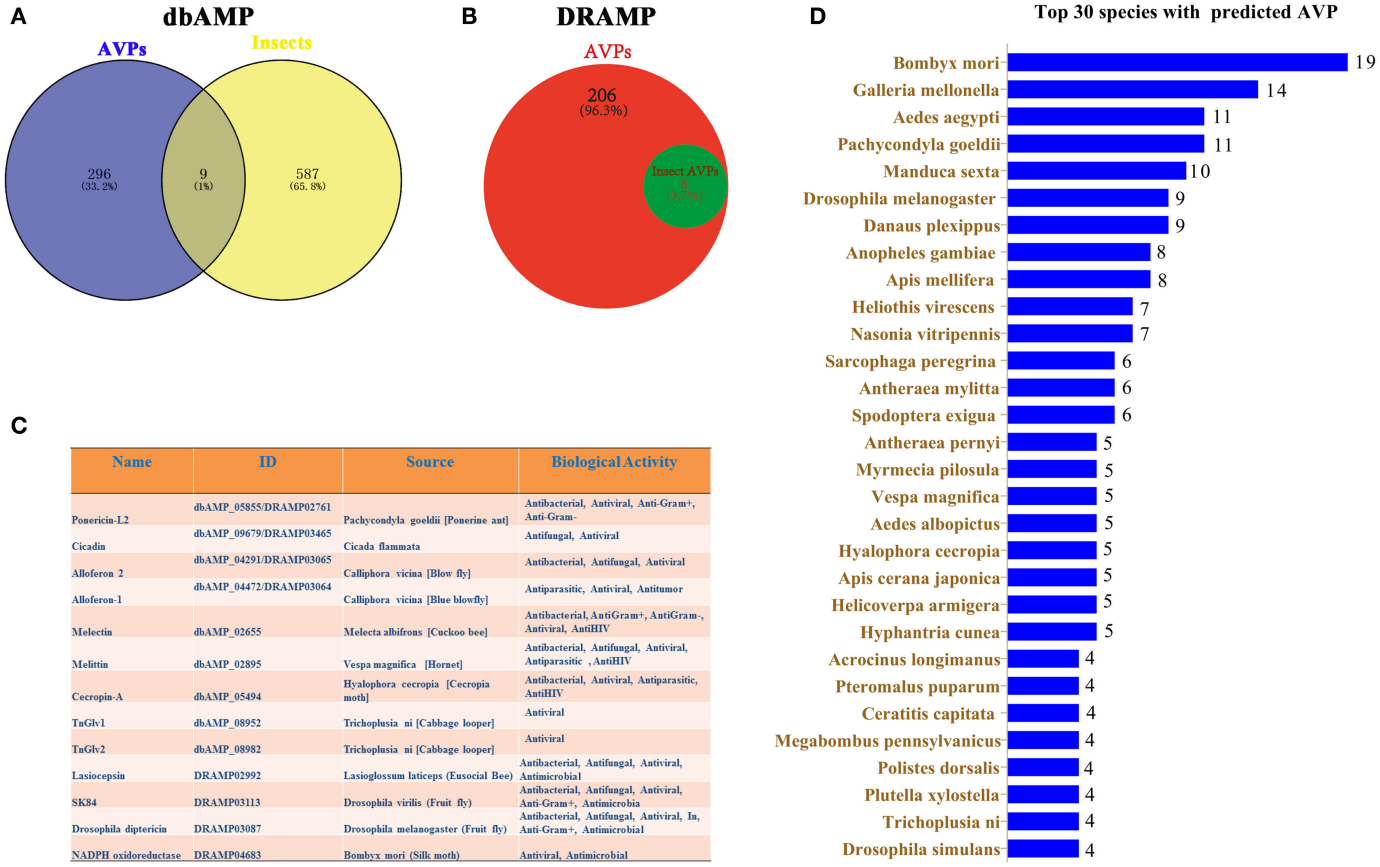
Prediction of insect AVPs from published databases. **(A)** The intersection between AMPs with potential antiviral activity and insect AMPs in the dbAMP database. **(B)** Insect AMPs with predicted antiviral activity in the DRAMP 2.0 database. **(C)** Integration of information on predicted insect AVPs from the dbAMP and DRAMP 2.0 databases. **(D)** Top 30 insects with predicted AVPs that were identified using Meta-iAVP.

The dbAMP was recently created as a useful resource for accumulating synthetic and natural AMPs from public AMP databases and scientific literature (34). In the dbAMP database, a total of 305 AVPs and 596 insect AMPs are collected (Figure 1A). Nine insect AVPs were obtained from the intersection of these two data sets in the dbAMP (Figure 1A). DRAMP 2.0 is an open-access comprehensive database containing general, patented and clinical AMPs (33). From this database, we identified 8 insect AVPs from a total 214 AVPs (Figure 1B). Integrating the insect AVPs information from the dbAMP and DRAMP 2.0 database, we obtained a total of 13 insect AVPs, which are shown in Figure 1C. Among hundreds of insect AMPs in the database, only 13 were associated with antiviral activity, which suggests that the research on insect AVP is still in in its infancy and requires more data. It can be assumed that many insect AMPs need to be explored for potential antiviral activity. Thus, the 596 insect AMPs in dbAMP database were further used to predict antiviral activity using Meta-iAVP (37). Unexpectedly, 392 insect AMPs were predicted as AVPs (predicted value >0.5) (Supplementary File 1). These predicted insect AVPs originated from *B. mori, Galleria mellonella, Aedes aegypti, Pachycondyla goeldii* (Ponerine ant), *Manduca sexta, D. melanogaster, Danaus plexippus, Anopheles gambiae, Apis mellifera* and others (Figure 1D). Based on this evidence, we have reason to believe that insect AMPs are a potential source for identification of AVPs, which is worthy of more in-depth study. However, at present, there is no special insect AMP database that can incorporate the latest review articles of insect AVPs. The existing databases continue to have omissions unless the information also becomes curated by professional insect researchers.

## Insect AMPS With Antiviral Activities: The Insect AVPS in Published Literature

Although the study of insect AVP as an important part of insect antiviral research was promoted more than 10 years ago (29), the available literature is still very limited. Surprisingly, until recently, few insect-derived AMPs were reported with documented antiviral activity. As shown in Table 2, ten insect AVPs were found to be involved in the antiviral response and the antiviral action was directed against both mammalian and insect viruses.

**Table 2:** Insect AVP reported in the literature.

**Insect AVP**	**Organism**	**Virus**	**References**
Cecropin-A	*H. cecropia*	HSV-1/ HIV-1/ JV	(39, 40)
Melittin	*A. mellifera*	HSV-1/HIV-1/JV/ influenza A viruses/ RSV/VSV/TMV/ enterovirus/ coxsackievirus	(39, 40)
Alloferon 1	*C. vicina*	Influenza viruses A/B/ HHV-1	(28)
Alloferon 2	*C. vicina*	Influenza viruses A/B	(28)
Myristoylated-peptide	*H. virescens*	HIV-1/HSV-1	(41)
TnGlv1	*T. ni*	AcMNPV	(42)
TnGlv2	*T. ni*	AcMNPV	(42)
attC	*Drosophila*	SINV	(23)
dptB	*Drosophila*	SINV	(23)
C-lysozyme	*B. mori*	BmNPV	(24)

Cecropin-A was one of the first animal antimicrobial peptides to be isolated and fully characterized from the hemolymph of the moth *Hyalophora cecropia* (43, 44). Subsequent research confirmed that Cecropin-A has inhibitory activity against human immunodefciency virus 1 (HIV-1; *Retroviridae*), herpes simplex virus 1 and 2 (HSV; *Herpesviridae*) and against the arenavirus Junin virus (JV) (39, 40).

Melittin belongs to the class of bee venom-derived AVPs and was isolated from the honeybee *A. mellifera* (45). This AVP was also tested against HSV, HIV-1 and JV, showing inhibition of viral replication for all tested viruses (40, 46). In addition, melittin also curbs infectivity of a diverse array of viruses including Coxsackie Virus and other enteroviruses (*Picornaviridae*), Influenza A viruses (*Orthomyxoviridae*), Respiratory Syncytial Virus (RSV; *Pneumoviridae*), Vesicular Stomatitis Virus (VSV; *Rhabdoviridae*) and the plant virus tobacco mosaic virus (TMV; *Virgaviridae*) (47). More information about the antiviral activity of melittin can be found in a review by Memariani et al. (47).

The insect AMP alloferon 1 and 2, derived from the hemolymph of blow fly *Calliphora vicina*, showed antiviral activity against influenza virus A and influenza virus B (28). Additional research also found that alloferon 1 inhibits human herpes virus type 1 (HHV-1; *Herpesviridae*) and analogs were active against coxsackievirus *in vitro* using cell lines (48, 49). Despite the mechanism of antiviral activity of alloferon is still unknown, Alloferon 1 and its analogs are considered as promising candidates for the design of new AVPs (50).

The antiviral compound N-myristoylated-peptide containing only six amino acids with molecular weight of 916 Da was purified from larval hemolymph of the tobacco budworm *Heliothis virescens* (41). Insect myristoylated-peptide has been confirmed to be effective against HIV-1 and HSV-1 (41). The N-terminus of N-myristoylated-peptide contains the fatty acid myristoyl and the C-terminus contains histidine with two methyl groups giving the histidine a permanent positive charge (41). The structure of the antiviral compound resembles the “myristate plus basic” motif present in particular viral proteins for binding to the cytoplasmic side of the plasma membrane to initiate virus assembly and budding from a host cell (41). It is speculated that the N-myristoylated-peptide is therefore able to specifically block or inhibit viruses like HIV-1 and HSV-1 that use this motif for exit from a host cell (41, 51).

Gloverin, a small cationic antibacterial protein, has been isolated from the hemolymph of various insects such as the giant silk moth *Hyalophora* (52) and the cabbage looper *Trichoplusia ni* (53). Two *T. ni* gloverin peptides named TnGlv1 and TnGlv2 showed resistance to the budded virus (BV) of *Autographa californica* multiple nucleopolyhedrovirus (AcMNPV; *Baculoviridae*) (42). The antiviral mechanism was speculated to be based on the accumulation of gloverin on the surface of BVs that may cause membrane strain or formation of pores that disrupt the BV envelope (42).

Two *Drosophila* AMP coding genes, diptericin B (dptB) and attacin C (attC), are upregulated in transgenic flies expressing a Sindbis virus (SINV) replicon. Silencing their expression led to a significant increase in SINV titers, suggesting that dptB and attC involved in *Drosophila* antiviral response to SINV (23). However, their mechanism of action remains to be elucidated.

Lysozyme is a ubiquitous peptide that is widely distributed in animals, plants, bacteria and viruses (54). The antibacterial, immunomodulatory and antiviral functions of lysozyme are well-known in vertebrates (55–57). More than fifty *lysozyme* genes have been identified from several insects (58), but the antiviral activity of insect lysozymes has not been widely investigated. In a recent study, the overexpression of *C-lysozyme* of *B. mori* could reduce *B. mori* nucleopolyhedrovirus (BmNPV) production and progeny virus virulence *in vivo* and *in vitro* (24). Further research is required to elucidate the antiviral mechanism of lysozyme peptides.

## Potential AVPS in Fruit Fly, Honeybee and Silkworm

Insects are the most speciose group of animals that inhabit almost all ecosystems and habitats on the land (17, 59). Although insects are a rich source of natural AMPs (17), only few insect AMPs have been confirmed with antiviral activity (Figure 1C, Table 2). In this study we have predicted 392 potential AVPs from 596 insect AMPs in the dbAMP database (Figure 1D, Supplementary File 1). This information may stimulate researchers to carry out in-depth and extensive research on the activity of the predicted insect AVPs. Insects, especially *D. melanogaster*, has been widely used as model for the study of innate immunity and microbial pathogens and for assessing the *in vivo* efficacy of antimicrobial agents (60). The silkworm and honeybee are well-known representative economic insects. In the following section, we will elaborate on potential AVPs in the fruit fly *D. melanogaster*, the two honeybee species *A. mellifera* and *A. cerana* and the silkworm *B. mori*.

### D. melanogaster

In general, seven well-characterized families including 21 inducible AMP/AMP-like genes have been identified in *Drosophila* (61, 62). The functions of *Drosophila* AMPs are not only involved in host defense, but expand also to gut microbiota homeostasis, tumor control, lifespan regulation and neurological processes (62, 63). However, to our knowledge, only two *Drosophila* AMPs, attC and dptB, have been reported to have antiviral function (23). Since the first animal AMP was discovered in insects (44), *D. melanogaster* has emerged as a powerful model for their characterization. Unfortunately, the research on antiviral immunity involving *Drosophila* AMPs has not received enough attention. After downloading the latest updated *Drosophila* AMP/AMP-like genes (including lysozyme) and their corresponding peptides from the NCBI database, their antiviral activity was predicted using Meta-iAVP (37). For AMP genes for which the mature peptide sequence was not determined, SignalP-5.0 was employed to predict the signal peptide and mature peptide (38).

Following this procedure, as shown in Table 3, a total of 23 potential AVPs were identified in *D. melanogaster*. We further analyzed these potential AVPs for their induction by viral infection in published transcriptome studies. Expression of *Defensin, Cecropin A1, Cecropin B, Andropin, Drosocin, Drosomycin, Metchnikowin, Lysozyme* S, *Attacin-B, Attacin*-*C, Diptericin A*, and *Lysozyme X* was found to be induced after viral infection in cell lines or adult flies (Table 3). Screening of transcriptome data for identification of key viral host factors is based on this concept (13). However, viruses may also interfere with the expression of antiviral factors as an immune escape strategy. Determination of antiviral activity based by induction of expression during viral infection is only indicative and cannot be considered as conclusive. But for screening of antiviral genes it can turn out to be a simple and effective method. Therefore, AMPs/AVPs that are up-regulated by a specific virus may be relatively reliable candidate host antiviral factors, for which further verification experiments have to be performed. It should also be noted that dptB has been shown to inhibit SINV replication (23), but it is not among the predicted candidate AVPs (Table 3). Thus, a strategy that screens virus-inducible genes clearly will not identify all potential AVPs.

**Table 3:** Predicted AVPs in *Drosophila*.

**Predicted AVP**	**Gene ID**	**Peptide ID**	**Value/precursor**	**Value/mature**	**Up-regulated by virus**
Defensin	36047	NP_523672.1	0.524	1	DCV (64, 65), DXV (64)
Cecropin A1	43596	NP_524588.1	0.908	0.946	DCV (66, 67), Sigma virus (64), CrPV (68)
Cecropin A2	43597	NP_524589.1	0.908	0.64	
Cecropin C	43599	NP_524591.1	1	0.744	
Cecropin B	43598	NP_524590.1	1	1	DCV (67)
Andropin	43595	NP_524587.1	0.762	0.524	DCV (67), FHV (69)
Drosocin	36635	NP_001246324.1/NP_523744.1	1	0.508	DXV (70), Sigma Virus (64)
Drosomycin	38419	NP_523901.1	0.992	0.524	DCV (64, 65, 71), DXV (64)
Drosomycin-like 5	38409	NP_647803.1	1	0.716	
Drosomycin-like 2	38408	NP_728860.2	1	0.946	
Drosomycin-like 3	317955	NP_728861.1	1	0.954	
Drosomycin-like 6	38416	NP_728873.1	0.92	0.892	
Drosomycin-like 1	326207	NP_728872.1	0.928	0.668	
Metchnikowin	36708	NP_523752.1	1	0.962	DCV (64, 65, 67, 71), DXV (64), SINV (23), CrPV (68)
Lysozyme P	38129	NP_476828.1	0.43(Non-AVP)	0.966	
Lysozyme S	38130	NP_476829.1	0.93	0.892	DCV (64), CrPV (68)
Attacin-B	36637	NP_001163152.1	0.64	0.07(Non-AVP)	DCV (66, 71), DXV (70), Sigma Virus (64), FHV (71), CrPV (68)
Attacin-C	36484	NP_523729.3	0.616	0(Non-AVP)	DCV (67, 71), SINV (23), FHV (71), CrPV (68)
Diptericin A	37183	NP_476808.1	0.86	0(Non-AVP)	Sigma Virus (64), CrPV (68)
Lysozyme B	38125	NP_001261245.1	0.986	0.282(Non-AVP)	
Lysozyme X	38122	NP_523881.1	0.774	0.272(Non-AVP)	FHV (71)
Lysozyme E	38128	NP_476827.2	1	0.008(Non-AVP)	

In addition, some non-classical AMPs such as Bomanins (72), Daishos (73) and Listericin (74) in *Drosophila* have also attracted our attention. An effector peptide family encoded by twelve *Bomanin* (*Bom*) genes has been found to be essential for effective *Drosophila* Toll-mediated immune responses (72). Daisho peptides, a new class of innate immune effectors in *Drosophila*, were recently found to have humoral activity against a set of filamentous fungi (73). Currently, these *Drosophila* peptides have not been confirmed to have antiviral activity. Using Meta-iAVP (37) prediction, we found that BomS1, BomS4, BomS6, BomT1, BomBc2, and Listericin have potential AVPs activity (Supplementary File 2).

### *A. mellifera* and *A. cerana*

Honeybees are important plant pollinators in both natural and agricultural ecosystems (75). Through pollination of flowering plants, honeybees do not only help to maintain biodiversity but in addition they also supply commodities such as honey, royal jelly, propolis (bee glue), pollen and wax. Viruses are significant threats to the health and well-being of the honeybee (76). Due to the abundance and economic importance of the honeybee, research on the interaction with bee viruses has received a lot of research interest. Honeybee antiviral defense mechanisms include RNAi, endocytosis, melanization, encapsulation, autophagy, pathogen-associated molecular pattern (PAMP)-triggered signal transduction cascades, and generation of reactive oxygen species (7, 77). There is currently no evidence that AMPs are involved in the antiviral response of honeybees (7, 77). However, melittin, the principal constituent in the venom of *A. mellifera*, has been demonstrated to be effective against the infectivity of a diverse array of mammalian viruses such as HIV and HSV (47). Venom-derived AMPs may not play a role in the antiviral response of its host, but the results of the antiviral experiments *in vitro* are an important reference of which the significance is not clear yet.

Following infection by pathogens, AMPs of four families comprising apidaecins (78), abaecins (79), hymenoptaecins (80), and defensins (81) are synthesized, representing a broad spectrum of antimicrobial activity in the haemolymph. Detailed comparison of these four AMP gene families between *A. mellifera* and *A. cerana* revealed that there are many similarities in the number and amino acid composition of the peptides in the abaecin, defensing, and apidaecin families, while many more hymenoptaecin peptides are found in *A. cerana* than in *A. mellifera* (19). Compared to *A. mellifera* that has a longer history of domestication, selection on *A. cerana* has favored the generation of more variable AMPs as protection against pathogens (19).

Using the predictive tools of Meta-iAVP (37), a total of 7 and 16 AVPs were obtained from *A. mellifera* and *A. cerana*, respectively (Table 4). Potential AVP genes of *A. mellifera* such as *defensin 1, defensin 2, abaecin, apisimin, hymenoptaecin*, and *lysozyme 3* were found to be up-regulated after infection with viruses such as Deformed wing virus (DWV), Sacbrood virus (SBV), black queen cell virus (BQCV), and Israeli acute paralysis virus (IAPV) in transcriptome data (Table 4). Almost all honeybee transcriptome studies that analyze virus infection are restricted to *A. mellifera* while little related research has been conducted on *A. cerana*. Recent research found that in *A. cerana* the predicted AVP genes *abaecin* and *hymenoptaecin* were significantly upregulated by Chinese Sacbrood virus (CSBV) infection (85). These potential AVPs, which are up-regulated by a specific honeybee virus, are important leads for future research on the antiviral immunity of honeybee AMPs.

**Table 4:** Predicted AVPs in *A. mellifera* and *A. cerana*.

**Predicted AVP/*A. mellifera***	**Gene ID (NCBI)**	**Peptide ID**	**Value/precursor**	**Value/mature**	**Up-regulated by virus**
Defensin 1	406143	NP_001011616.2	0.966	0.772	DWV+SBV (82)
Defensin 2	413397	NP_001011638.1	0.916	0.43 (Non-AVP)	DWV+SBV (82)
Abaecin	406144	NP_001011617.1	1	0.64	DWV+SBV (82), BQCV (83)
Apisimin	406093	NP_001011582.1	0.586	0.974	DWV+SBV (82)
Hymenoptaecin	406142	NP_001011615.1	0.282 (Non-AVP)	0.542	DWV+SBV (82), IAPV (84), BQCV (83)
Lysozyme 1/2	724899	XP_026300526.1	0.078 (Non-AVP)	0.548	
lysozyme 3	409663	XP_393161.3	0.64	0.98	DWV+SBV (82)
***A. cerana***					
Defensin-2	108000415	XP_016916212.1	0.992	1	
Abaecin	108002218	XP_016919244.1	0.354 (Non-AVP)	0.906	CSBV (85)
Apidaecins type 22	108000468	XP_016916307.1	0.542	0.876	
Hymenoptaecin	107993492	XP_016905415.1	0.694	0 (Non-AVP)	CSBV (85)
Apisimin	108003250	XP_016920890.1	0.994	0.98	
AcDef7	EU727274	ACH96390.1	0.986	0.932	
AcHym3	EU727299	ACH96415.1	0.508	0.752	
AcHym16	EU727312	ACH96428.1	0.104 (Non-AVP)	0.536	
AcHym18	EU727314	ACH96430.1	0.696	0.028 (Non-AVP)	
AcHym1	EU727297	ACH96413.1	0.268 (Non-AVP)	0.696	
AcHym4	EU727300	ACH96416.1	0.716	0 (Non-AVP)	
AcHym7	EU727303	ACH96419.1	0.072 (Non-AVP)	0.876	
AcHym9	EU727305	ACH96421.1	0.694	0 (Non-AVP)	
AcHym25	EU835174	ACJ22829.1	0.508	0.752	
Lysozyme-like	108000169	XP_028523646.1	0.078 (Non-AVP)	1	
Lysozyme-like	114577830	XP_028523645.1	0.746	1	

### B. mori

The domestic silkworm *B. mori*, is an important lepidopteran insect of high scientific and economic value (86). Like in apiculture, the viral disease can cause enormous economic loss in sericulture (87). For viral diseases of silkworm, currently there is no effective treatment. Although there exist specific strains of silkworm that are resistant to some viruses, the specific mechanism is unclear (88–90). Like other insects, RNAi was considered as the major defense strategy against viral infections in *B. mori* (91). However, the antiviral innate immune response of silkworm has not been systematically studied although specific antiviral molecules such as PP2A (92), BmSTING (93), BmAtlastin-n (94), BmNOX (95), Bmlipase-1 (96), were identified. In a review article the involvement of AMPs in the antiviral response of silkworm was claimed (6), but in fact very few specific cases of antiviral activity of silkworm AMPs are known, an exception being a recent article on inhibition of BmNPV by lysozyme (24). Interestingly, a study reported that *B. mori* peptidoglycan recognition protein S2 (BmPGRP-S2) overexpression could activate the Imd pathway and induce AMP upregulation, enhancing silkworm antiviral resistance (97).

Following the publication of the genome of the silkworm (86), 35 silkworm AMP genes were identified based on the silkworm genome sequence and expressed sequence tags databases (20). These silkworm AMP genes belong to six families including cecropins, moricins, gloverins, attacins, enbocins, and lebocin (20). Following analysis of updated AMP gene data in the NCBI database, 21 potential silkworm AVPs (Table 5) were obtained using Meta-iAVP prediction (37). Among these potential AVP genes, *gloverin-2, gloverin-3, lebocin, attacin 1*, and *lysozyme* have been found to be induced by BmNPV infection in both resistant and susceptible silkworms (98, 100). It is worth noting that the expression of the potential AVP gene *gloverin-4* was significantly up-regulated only in BmNPV-infected resistant silkworm, while no changes were found in the BmNPV-infected susceptible silkworm and BmN cells, further suggesting that gloverin-4 is an AVP against BmNPV infection (98). The expression of the potential AVP gene *cecropin A* and *cecropin B* also tended to be up-regulated during infection with *B. mori* cytoplasmic polyhedrosis virus (BmCPV), but expression levels were too low to be considered as biologically important (99). Moreover, it is curious that although many omics data related to silkworm virus infection have been published, no more clues were obtained about the involvement of AMPs in the defense against *B. mori* bidensovirus (BmBDV), BmNPV and BmCPV infection (101–105).

**Table 5:** Predicted AVPs in *B. mori*.

**Predicted AVP**	**Gene ID**	**Peptide ID**	**Value/precursor**	**Value/mature**	**Up-regulated by virus**
Attacin1	692555	NP_001037006.1	0.936	0.044 (Non-AVP)	BmNPV (98)
Attacin-like	101743224	XP_004926758.1	0.726	0.986	
Cecropin B	732858	NP_001096031.1	1	0.992	BmCPV (99)
Cecropin A	693029	NP_001037462.1	1	0.964	BmCPV (99)
Cecropin-like	101739821	NP_001037392.1	0.962	0.998	
Cecropin-D-like peptide	101740228	NP_001036924.2	1	0.694	
Cecropin D	692369	NP_001036833.1	0.988	0.892	
Cecropin CBM2	692583	NP_001037031.1	0.536	0.97	
Defensin	692778	NP_001037370.1	0.982	0.924	
Enbocin1	693035	NP_001037472.1	0.982	0.616	
Enbocin3	100101217	NP_001093310.1	0.854	0.998	
Gloverin 2	692527	NP_001037683.1	0.668	0.506	BmNPV (100)
Gloverin 3	692476	NP_001093312.1	0.068 (Non-AVP)	0.678	BmNPV (98, 100)
Gloverin 4	751090	NP_001037684.1	0.07 (Non-AVP)	0.81	BmNPV (98, 100)
Gloverin 4-like	692477	NP_001036932.1	0.038 (Non-AVP)	1	
Lebocin	100146108	NP_001119732.2	0.536	0.164 (Non-AVP)	BmNPV (98, 100)
Moricin	692365	NP_001036829.2	0.992	0.964	
Moricin-1-like	105842862	XP_012552566.1	0.536	0.908	
Moricin-1-like	101742278	XP_012551343.2	0.996	0.908	
Moricin-1-like	101742127	XP_012551345.2	0.554	0.818	
Lysozyme	693015	NP_001037448.1	0.968	0.678	BmNPV (100)

## The Program of AVP Synthesis and its Mechanism of Action in Insects

Universally, after the virus invades the host, the host will initiate a recognition mechanism and induce a downstream antiviral cascade reaction. In vertebrates, during various viral infections, virus-associated PAMPs are recognized by pathogen recognition receptors (PRRs) such as Toll-like receptors (TLRs), retinoic acid-inducible gene I (RIG-I)-like receptors (RLRs), NOD-like receptors (12), interferon-γ-inducible protein 16 (IFI16), AIM2 (absent in melanoma 2) and cyclic GMP-AMP synthase (cGAS) that subsequently lead to the activation of inflammatory cytokines and chemokines as well as interferon (IFN) and ISG production through a cascade reaction (106). However, similar antiviral response systems have not been systematically studied in insects. At present, we have very limited knowledge of how insects recognize virus invasion and initiate cascade reactions to exert antiviral functions.

**Figure 2:**
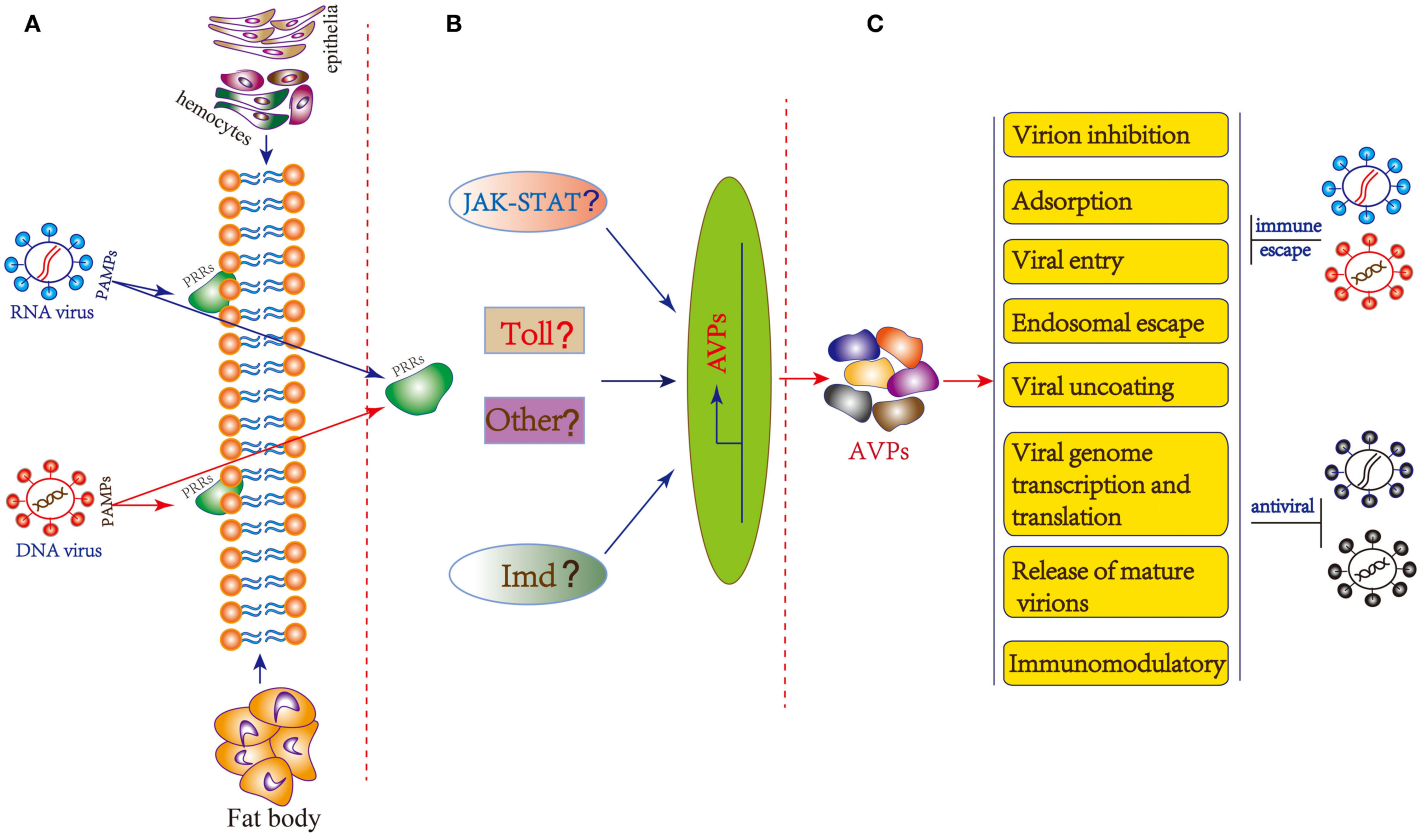
General hypothesis of AVP synthesis and possible mechanism of action in insects. **(A)** Immune recognition of insect viruses. The PAMPs of insect RNA and DNA viruses are recognized by specific PRRs located in the cell membrane or cytoplasm of hemocytes, epithelia or fat body. **(B)** Potential downstream signaling cascade reactions including JAK-STAT, Toll, Imd, and other pathway to produce AVPs. **(C)** The mechanism of action of AVPs covers stages in almost the entire life cycle of the virus: virion inhibition; adsorption; viral entry; endosomal escape; viral uncoating; viral genome transcription and translation, and release of mature virions. Additionally, AVPs may inhibit viral infection by regulating the host immune system. As a counterdefense, insect viruses may employ several strategies to escape the antiviral effect of AVPs.

In insects, a number of actual and potential PRRs such as TLRs, peptidoglycan recognition proteins (PGRPs), Gram-negative bacteria-binding proteins (GNBPs), scavenger receptors (SRs), thioester-containing proteins (TEPs) and lectins have been identified (107, 108). Unfortunately, there is currently no evidence that any of the above-mentioned PRRs are involved in insect virus recognition, with the exception of the nucleic acid sensor Dicer-2 that can act as a PRR of double-stranded RNA in parallel to the RNAi pathway (107). Recently, *B. mori* cGAMP and PGRP2 were confirmed to be involved in host responses to BmNPV (93, 109). In *Drosophila*, Toll, IMD and JAK/STAT pathway may be involved in antiviral immunity (4, 65, 110). In addition, JAK/STAT pathway could also be activated by challenge with BmNPV and BmBDV (111). The classical innate immune pathways are also transcriptionally induced during pathogenic infection of Bm5 cells with RNA virus (112). However, the insect PRRs for viral recognition and signaling pathway activation have not been fully elucidated. Thus, there is currently no exact mechanism identified for the generation of AVPs and more in-depth research is needed. Based on evidence obtained in vertebrate (mammalian) systems, we can make the hypothesis that insect viral PAMPs are recognized by specific PRRs located in the cell membrane or cytoplasm of hemocytes, epithelia or fat body which then triggers downstream signaling cascades for the production of AVPs (Figure 2).

The AVPs possess diverse structures as well as might act according to different mechanisms. Based on the antiviral peptide database AVPdb (Table 1), a total of 45 virus targeting strategies employed by AVPs can be distinguished such as “Virus entry,” “Virucidal on progeny virions,” “Viral assembly,” “Release,” “Transcription,” “Translation,” “Transport,” and “Replication” (31). The mechanism of action of AVPs summarized in the AVPdb database covers almost the entire life cycle of the virus (Figure 2). Additionally, AVPs may act against viral infection by regulating the host immune system (Figure 2). For instance AVP like alloferons from the blow fly are able to stimulate natural killer cells (NK) activity and interferon synthesis in animal and human models (28).

## Future Research

Many scientific questions about the identities of insect AVPs and their modes of action remain unresolved. Besides, viruses are the causative agents of various dreadful diseases in humans and animals. Recently, the testing and discovery of AVPs was accelerated because extraordinary advantages. Insects are considered an important source of natural AMPs, and their potential to act as AVPs is worthy of in-depth studies. In future research, the research on insect AVPs can mainly focus on the following key issues: (1) Identification of insect AVPs; (2) Recognition by PRRs and downstream cascade reactions involved in insect AVPs production; (3) Molecular mechanism of action of AVPs against insect viruses and vertebrate viruses; (4) AVP counter defense (immune escape) mechanisms by viruses; (5) Evaluation and application of insect AVPs as antiviral drugs.

## Author Contributions

MF participated in the design, collected and analyzed data, and drafted the manuscript. SF and JX helped with data collection. VL, LS, and JS participated in the design and coordination of the study, and revised the manuscript. All authors read and approved the final manuscript.

## Conflict of Interest

The authors declare that the research was conducted in the absence of any commercial or financial relationships that could be construed as a potential conflict of interest.

## Supplementary Material

Supplementary File 1 Antiviral activity prediction of all insect AMPs in the dbAMP database.

Supplementary File 2 Antiviral activity prediction of Bomanins, Daishos, and Listericin in *Drosophila*.

## References

[1] Lemaitre B Nicolas E Michaut L Reichhart JM Hoffmann JA. The dorsoventral regulatory gene cassette spatzle/Toll/cactus controls the potent antifungal response in Drosophila adults. Cell. (1996) 86:973–83. 10.1016/S0092-8674(00)80172-5 8808632

[2] Hoffmann JA Kafatos FC Janeway CA Ezekowitz RA. Phylogenetic perspectives in innate immunity. Science. (1999) 284:1313–8. 10.1126/science.284.5418.1313 10334979

[3] Mussabekova A Daeffler L Imler JL. Innate and intrinsic antiviral immunity in *Drosophila*. Cell Mol Life Sci. (2017) 74:2039–54. 10.1007/s00018-017-2453-9 28102430 PMC5419870

[4] Xu J Cherry S. Viruses and antiviral immunity in *Drosophila*. Dev Comp Immunol. (2014) 42:67–84. 10.1016/j.dci.2013.05.002 23680639 PMC3826445

[5] Swevers L Liu J Smagghe G. Defense mechanisms against viral infection in *Drosophila*: RNAi and Non-RNAi. Viruses. (2018) 10:230. 10.3390/v10050230 29723993 PMC5977223

[6] Lu P Pan Y Yang Y Zhu F Li C Guo Z. Discovery of anti-viral molecules and their vital functions in *Bombyx mori*. J Invertebr Pathol. (2018) 154:12–8. 10.1016/j.jip.2018.02.012 29453967

[7] McMenamin AJ Daughenbaugh KF Parekh F Pizzorno MC Flenniken ML. Honey bee and bumble bee antiviral defense. Viruses. (2018) 10:395. 10.3390/v10080395 30060518 PMC6115922

[8] Chen H Jiang Z. The essential adaptors of innate immune signaling. Protein Cell. (2013) 4:27–39. 10.1007/s13238-012-2063-0 22996173 PMC4875439

[9] Yan N Chen ZJ. Intrinsic antiviral immunity. Nat Immunol. (2012) 13:214–22. 10.1038/ni.2229 22344284 PMC3549670

[10] Schoggins JW. Interferon-stimulated genes: roles in viral pathogenesis. Curr Opin Virol. (2014) 6:40–6. 10.1016/j.coviro.2014.03.006 24713352 PMC4077717

[11] Schoggins JW Wilson SJ Panis M Murphy MY Jones CT Bieniasz P. A diverse range of gene products are effectors of the type I interferon antiviral response. Nature. (2011) 472:481–5. 10.1038/nature09907 21478870 PMC3409588

[12] Liu SY Sanchez DJ Aliyari R Lu S Cheng G. Systematic identification of type I and type II interferon-induced antiviral factors. Proc Natl Acad Sci USA. (2012) 109:4239–44. 10.1073/pnas.1114981109 22371602 PMC3306696

[13] Xie T Feng M Dai M Mo G Ruan Z Wang G. Cholesterol-25-hydroxylase is a chicken ISG that restricts ALV-J infection by producing 25-hydroxycholesterol. Viruses. (2019) 11:498. 10.3390/v11060498 31151272 PMC6631237

[14] Wang X Li Y Li LF Shen L Zhang L Yu J. RNA interference screening of interferon-stimulated genes with antiviral activities against classical swine fever virus using a reporter virus. Antiviral Res. (2016) 128:49–56. 10.1016/j.antiviral.2016.02.001 26868874

[15] Levraud JP Jouneau L Briolat V Laghi V Boudinot P. IFN-stimulated genes in zebrafish and humans define an ancient arsenal of antiviral immunity. J Immunol. (2019) 203:3361–73. 10.4049/jimmunol.1900804 31732531

[16] Yi HY Chowdhury M Huang YD Yu XQ. Insect antimicrobial peptides and their applications. Appl Microbiol Biotechnol. (2014) 98:5807–22. 10.1007/s00253-014-5792-6 24811407 PMC4083081

[17] Sheehan G Farrell G Kavanagh K. Immune priming: the secret weapon of the insect world. Virulence. (2020) 11:238–46. 10.1080/21505594.2020.1731137 32079502 PMC7051127

[18] Imler JL Bulet P. Antimicrobial peptides in *Drosophila*: structures, activities and gene regulation. Chem Immunol Allergy. (2005) 86:1–21. 10.1159/000086648 15976485

[19] Xu P Shi M Chen XX. Antimicrobial peptide evolution in the Asiatic honey bee *Apis cerana*. PLoS ONE. (2009) 4:e4239. 10.1371/journal.pone.0004239 19156201 PMC2617784

[20] Cheng T Zhao P Liu C Xu P Gao Z Xia Q. Structures, regulatory regions, and inductive expression patterns of antimicrobial peptide genes in the silkworm *Bombyx mori*. Genomics. (2006) 87:356–65. 10.1016/j.ygeno.2005.11.018 16406194

[21] Liao JF Wu CP Tang CK Tsai CW Rouhova L Wu YL. Identification of regulatory host genes involved in sigma virus replication using RNAi knockdown in *Drosophila*. Insects. (2019) 10:339. 10.3390/insects10100339 31614679 PMC6835446

[22] Panda D Rose PP Hanna SL Gold B Hopkins KC Lyde RB. Genome-wide RNAi screen identifies SEC61A and VCP as conserved regulators of *Sindbis virus* entry. Cell Rep. (2013) 5:1737–48. 10.1016/j.celrep.2013.11.028 24332855 PMC3920290

[23] Huang Z Kingsolver MB Avadhanula V Hardy RW. An antiviral role for antimicrobial peptides during the arthropod response to alphavirus replication. J Virol. (2013) 87:4272–80. 10.1128/JVI.03360-12 23365449 PMC3624382

[24] Chen TT Tan LR Hu N Dong ZQ Hu ZG Jiang YM. C-lysozyme contributes to antiviral immunity in *Bombyx mori* against nucleopolyhedrovirus infection. J Insect Physiol. (2018) 108:54–60. 10.1016/j.jinsphys.2018.05.005 29778904

[25] Palmer WH Joosten J Overheul GJ Jansen PW Vermeulen M Obbard DJ. Induction and suppression of NF-kappaB signalling by a DNA virus of *Drosophila*. J Virol. (2019) 93:e01443–18. 10.1128/JVI.01443-18 30404807 PMC6340046

[26] Vilas Boas LCP Campos ML Berlanda RLA de Carvalho Neves N Franco OL. Antiviral peptides as promising therapeutic drugs. Cell Mol Life Sci. (2019) 76:3525–42. 10.1007/s00018-019-03138-w 31101936 PMC7079787

[27] Gentilucci L De Marco R Cerisoli L. Chemical modifications designed to improve peptide stability: incorporation of non-natural amino acids, pseudo-peptide bonds, and cyclization. Curr Pharm Des. (2010) 16:3185–203. 10.2174/138161210793292555 20687878

[28] Chernysh S Kim SI Bekker G Pleskach VA Filatova NA Anikin VB. Antiviral and antitumor peptides from insects. Proc Natl Acad Sci USA. (2002) 99:12628–32. 10.1073/pnas.192301899 12235362 PMC130511

[29] Slocinska M Marciniak P Rosinski G. Insects antiviral and anticancer peptides: new leads for the future? Protein Pept Lett. (2008) 15:578–85. 10.2174/092986608784966912 18680452

[30] Wang G Li X Wang Z. APD3: the antimicrobial peptide database as a tool for research and education. Nucleic Acids Res. (2016) 44:D1087–93. 10.1093/nar/gkv1278 26602694 PMC4702905

[31] Qureshi A Thakur N Tandon H Kumar M. AVPdb: a database of experimentally validated antiviral peptides targeting medically important viruses. Nucleic Acids Res. (2014) 42:D1147–53. 10.1093/nar/gkt1191 24285301 PMC3964995

[32] Mehta D Anand P Kumar V Joshi A Mathur D Singh S. ParaPep: a web resource for experimentally validated antiparasitic peptide sequences and their structures. Database. (2014) 2014:bau051. 10.1093/database/bau051 24923818 PMC4054663

[33] Kang X Dong F Shi C Liu S Sun J Chen J. DRAMP 2.0, an updated data repository of antimicrobial peptides. Sci Data. (2019) 6:148. 10.1038/s41597-019-0154-y 31409791 PMC6692298

[34] Jhong JH Chi YH Li WC Lin TH Huang KY Lee TY. dbAMP: an integrated resource for exploring antimicrobial peptides with functional activities and physicochemical properties on transcriptome and proteome data. Nucleic Acids Res. (2019) 47:D285–97. 10.1093/nar/gky1030 30380085 PMC6323920

[35] Thakur N Qureshi A Kumar M. AVPpred: collection and prediction of highly effective antiviral peptides. Nucleic Acids Res. (2012) 40:W199–204. 10.1093/nar/gks450 22638580 PMC3394244

[36] Beltran Lissabet JF Belen LH Farias JG. AntiVPP 1.0: a portable tool for prediction of antiviral peptides. Comput Biol Med. (2019) 107:127–30. 10.1016/j.compbiomed.2019.02.011 30802694 PMC7094449

[37] Schaduangrat N Nantasenamat C Prachayasittikul V Shoombuatong W. Meta-iAVP: a sequence-based meta-predictor for improving the prediction of antiviral peptides using effective feature representation. Int J Mol Sci. (2019) 20:5743. 10.3390/ijms20225743 31731751 PMC6888698

[38] Almagro Armenteros JJ Tsirigos KD Sonderby CK Petersen TN Winther O Brunak S SignalP 5.0 improves signal peptide predictions using deep neural networks. Nat Biotechnol. (2019) 37:420–3. 10.1038/s41587-019-0036-z 30778233

[39] Wachinger M Kleinschmidt A Winder D von Pechmann N Ludvigsen A Neumann M. Antimicrobial peptides melittin and cecropin inhibit replication of human immunodeficiency virus 1 by suppressing viral gene expression. J Gen Virol. (1998) 79:731–40. 10.1099/0022-1317-79-4-731 9568968

[40] Albiol Matanic VC Castilla V. Antiviral activity of antimicrobial cationic peptides against Junin virus and herpes simplex virus. Int J Antimicrob Agents. (2004) 23:382–9. 10.1016/j.ijantimicag.2003.07.022 15081088

[41] Ourth DD. Antiviral activity against human immunodeficiency virus-1 *in vitro* by myristoylated-peptide from *Heliothis virescens*. Biochem Biophys Res Commun. (2004) 320:190–6. 10.1016/j.bbrc.2004.05.137 15207720

[42] Moreno-Habel DA Biglang-awa IM Dulce A Luu DD Garcia P Weers PM. Inactivation of the budded virus of *Autographa californica* M nucleopolyhedrovirus by gloverin. J Invertebr Pathol. (2012) 110:92–101. 10.1016/j.jip.2012.02.007 22401766 PMC3327827

[43] Hultmark D Steiner H Rasmuson T Boman HG. Insect immunity. Purification and properties of three inducible bactericidal proteins from hemolymph of immunized pupae of *Hyalophora cecropia*. Eur J Biochem. (1980) 106:7–16. 10.1111/j.1432-1033.1980.tb05991.x 7341234

[44] Steiner H Hultmark D Engstrom A Bennich H Boman HG. Sequence and specificity of two antibacterial proteins involved in insect immunity. Nature. (1981) 292:246–8. 10.1038/292246a0 7019715

[45] Bazzo R Tappin MJ Pastore A Harvey TS Carver JA Campbell ID. The structure of melittin. A 1H-NMR study in methanol. Eur J Biochem. (1988) 173:139–46. 10.1111/j.1432-1033.1988.tb13977.x 3356186

[46] Wachinger M Saermark T Erfle V. Influence of amphipathic peptides on the HIV-1 production in persistently infected T lymphoma cells. FEBS Lett. (1992) 309:235–41. 10.1016/0014-5793(92)80780-K 1516693

[47] Memariani H Memariani M Moravvej H Shahidi-Dadras M. Melittin: a venom-derived peptide with promising anti-viral properties. Eur J Clin Microbiol Infect Dis. (2020) 39:5–17. 10.1007/s10096-019-03674-0 31422545 PMC7224078

[48] Kuczer M Dziubasik K Midak-Siewirska A Zahorska R Luczak M Konopinska D. Studies of insect peptides alloferon, Any-GS and their analogues. Synthesis and antiherpes activity. J Pept Sci. (2010) 16:186–9. 10.1002/psc.1219 20191606

[49] Kuczer M Midak-Siewirska A Zahorska R Luczak M Konopinska D. Further studies on the antiviral activity of alloferon and its analogues. J Pept Sci. (2011) 17:715–19. 10.1002/psc.1388 21766388

[50] Kuczer M Majewska A Zahorska R. New alloferon analogues: synthesis and antiviral properties. Chem Biol Drug Des. (2013) 81:302–9. 10.1111/cbdd.12020 22883213

[51] Resh MD. Fatty acylation of proteins: new insights into membrane targeting of myristoylated and palmitoylated proteins. Biochim Biophys Acta. (1999) 1451:1–16. 10.1016/S0167-4889(99)00075-0 10446384

[52] Axen A Carlsson A Engstrom A Bennich H. Gloverin, an antibacterial protein from the immune hemolymph of *Hyalophora pupae*. Eur J Biochem. (1997) 247:614–9. 10.1111/j.1432-1033.1997.00614.x 9266704

[53] Lundstrom A Liu G Kang D Berzins K Steiner H. *Trichoplusia ni* gloverin, an inducible immune gene encoding an antibacterial insect protein. Insect Biochem Mol Biol. (2002) 32:795–801. 10.1016/S0965-1748(01)00162-X 12044496

[54] Gajda E Bugla-Ploskonska G. Lysozyme–occurrence in nature, biological properties and possible applications. Postepy Hig Med Dosw. (2014) 68:1501–15. 10.5604/17322693.1133100 25531714

[55] Ragland SA Criss AK. From bacterial killing to immune modulation: recent insights into the functions of lysozyme. PLoS Pathog. (2017) 13:e1006512. 10.1371/journal.ppat.1006512 28934357 PMC5608400

[56] Singh IP Bodiwala HS. Recent advances in anti-HIV natural products. Nat Prod Rep. (2010) 27:1781–800. 10.1039/c0np00025f 20976350

[57] Villa TG Feijoo-Siota L Rama JLR Ageitos JM. Antivirals against animal viruses. Biochem Pharmacol. (2017) 133:97–116. 10.1016/j.bcp.2016.09.029 27697545 PMC7092833

[58] Mohamed AA Zhang L Dorrah MA Elmogy M Yousef HA Bassal TT. Molecular characterization of a c-type lysozyme from the desert locust, *Schistocerca gregaria* (Orthoptera: acrididae). Dev Comp Immunol. (2016) 61:60–9. 10.1016/j.dci.2016.03.018 26997372

[59] Misof B Liu S Meusemann K Peters RS Donath A Mayer C. Phylogenomics resolves the timing and pattern of insect evolution. Science. (2014) 346:763–7. 10.1126/science.1257570 25378627

[60] Yamaguchi M Yoshida H. *Drosophila* as a model organism. Adv Exp Med Biol. (2018) 1076:1–10. 10.1007/978-981-13-0529-0_1 29951811

[61] Hetru C Troxler L Hoffmann JA. *Drosophila melanogaster* antimicrobial defense. J Infect Dis. (2003) 187(Suppl. 2):S327–34. 10.1086/374758 12792847

[62] Hanson MA Lemaitre B. New insights on *Drosophila* antimicrobial peptide function in host defense and beyond. Curr Opin Immunol. (2020) 62:22–30. 10.1016/j.coi.2019.11.008 31835066

[63] Loch G Zinke I Mori T Carrera P Schroer J Takeyama H. Antimicrobial peptides extend lifespan in *Drosophila*. PLoS ONE. (2017) 12:e0176689. 10.1371/journal.pone.0176689 28520752 PMC5435158

[64] Tsai CW McGraw EA Ammar ED Dietzgen RG Hogenhout SA. *Drosophila melanogaster* mounts a unique immune response to the *Rhabdovirus sigma* virus. Appl Environ Microbiol. (2008) 74:3251–6. 10.1128/AEM.02248-07 18378641 PMC2394955

[65] Dostert C Jouanguy E Irving P Troxler L Galiana-Arnoux D Hetru C. The Jak-STAT signaling pathway is required but not sufficient for the antiviral response of drosophila. Nat Immunol. (2005) 6:946–53. 10.1038/ni1237 16086017

[66] Roxstrom-Lindquist K Terenius O Faye I. Parasite-specific immune response in adult *Drosophila melanogaster*: a genomic study. EMBO Rep. (2004) 5:207–12. 10.1038/sj.embor.7400073 14749722 PMC1298984

[67] Zhu F Ding H Zhu B. Transcriptional profiling of *Drosophila* S2 cells in early response to *Drosophila* C virus. Virol J. (2013) 10:210. 10.1186/1743-422X-10-210 23803447 PMC3704779

[68] Merkling SH Overheul GJ van Mierlo JT Arends D Gilissen C van Rij RP. The heat shock response restricts virus infection in *Drosophila*. Sci Rep. (2015) 5:12758. 10.1038/srep12758 26234525 PMC4522674

[69] Castorena KM Stapleford KA Miller DJ. Complementary transcriptomic, lipidomic, and targeted functional genetic analyses in cultured *Drosophila* cells highlight the role of glycerophospholipid metabolism in flock house virus RNA replication. BMC Genom. (2010) 11:183. 10.1186/1471-2164-11-183 20236518 PMC2847973

[70] Zambon RA Nandakumar M Vakharia VN Wu LP. The toll pathway is important for an antiviral response in *Drosophila*. Proc Natl Acad Sci USA. (2005) 102:7257–62. 10.1073/pnas.0409181102 15878994 PMC1129099

[71] Kemp C Mueller S Goto A Barbier V Paro S Bonnay F. Broad RNA interference-mediated antiviral immunity and virus-specific inducible responses in *Drosophila*. J Immunol. (2013) 190:650–8. 10.4049/jimmunol.1102486 23255357 PMC3538939

[72] Clemmons AW Lindsay SA Wasserman SA. An effector peptide family required for *Drosophila* toll-mediated immunity. PLoS Pathog. (2015) 11:e1004876. 10.1371/journal.ppat.1004876 25915418 PMC4411088

[73] Cohen LB Lindsay SA Xu Y Lin SJH Wasserman SA. The daisho peptides mediate *Drosophila* defense against a subset of filamentous fungi. Front. Immunol. (2020) 11:9. 10.3389/fimmu.2020.00009 32038657 PMC6989431

[74] Goto A Yano T Terashima J Iwashita S Oshima Y Kurata S. Cooperative regulation of the induction of the novel antibacterial listericin by peptidoglycan recognition protein LE and the JAK-STAT pathway. J Biol Chem. (2010) 285:15731–8. 10.1074/jbc.M109.082115 20348097 PMC2871439

[75] Calderone NW. Insect pollinated crops, insect pollinators and US agriculture: trend analysis of aggregate data for the period 1992–2009. PLoS ONE. (2012) 7:e37235. 10.1371/journal.pone.0037235 22629374 PMC3358326

[76] Chen YP Siede R. Honey bee viruses. Adv Virus Res. (2007) 70:33–80. 10.1016/S0065-3527(07)70002-7 17765703

[77] Brutscher LM Daughenbaugh KF Flenniken ML. Antiviral defense mechanisms in honey bees. Curr Opin Insect Sci. (2015) 10:71–82. 10.1016/j.cois.2015.04.016 26273564 PMC4530548

[78] Casteels P Ampe C Jacobs F Vaeck M Tempst P. Apidaecins: antibacterial peptides from honeybees. EMBO J. (1989) 8:2387–91. 10.1002/j.1460-2075.1989.tb08368.x 2676519 PMC401180

[79] Casteels P Ampe C Riviere L Van Damme J Elicone C Fleming M. Isolation and characterization of abaecin, a major antibacterial response peptide in the honeybee (*Apis mellifera*). Eur J Biochem. (1990) 187:381–6. 10.1111/j.1432-1033.1990.tb15315.x 2298215

[80] Casteels P Ampe C Jacobs F Tempst P. Functional and chemical characterization of Hymenoptaecin, an antibacterial polypeptide that is infection-inducible in the honeybee (*Apis mellifera*). J. Biol. Chem. (1993) 268:7044–54. 8463238

[81] Casteels-Josson K Zhang W Capaci T Casteels P Tempst P. Acute transcriptional response of the honeybee peptide-antibiotics gene repertoire and required post-translational conversion of the precursor structures. J Biol Chem. (1994) 269:28569–75. 7961803

[82] Ryabov EV Fannon JM Moore JD Wood GR Evans DJ The iflaviruses sacbrood virus and deformed wing virus evoke different transcriptional responses in the honeybee which may facilitate their horizontal or vertical transmission. PeerJ. (2016) 4:e1591 10.7717/peerj.1591 26819848 PMC4727977

[83] Doublet V Paxton RJ McDonnell CM Dubois E Nidelet S Moritz RF. Brain transcriptomes of honey bees (*Apis mellifera*) experimentally infected by two pathogens: black queen cell virus and *Nosema ceranae*. Genom. Data. (2016) 10:79–82. 10.1016/j.gdata.2016.09.010 27747157 PMC5054260

[84] Galbraith DA Yang X Nino EL Yi S Grozinger C. Parallel epigenomic and transcriptomic responses to viral infection in honey bees (*Apis mellifera*). PLoS Pathog. (2015) 11:e1004713. 10.1371/journal.ppat.1004713 25811620 PMC4374888

[85] Shan L Liuhao W Jun G Yujie T Yanping C Jie W. Chinese sacbrood virus infection in Asian honey bees (*Apis cerana* cerana) and host immune responses to the virus infection. J Invertebr Pathol. (2017) 150:63–9. 10.1016/j.jip.2017.09.006 28916146

[86] Xia Q Zhou Z Lu C Cheng D Dai F Li B. A draft sequence for the genome of the domesticated silkworm (*Bombyx mori*). Science. (2004) 306:1937–40. 10.1126/science.1102210 15591204

[87] Swevers L Feng M Ren F Sun J. Antiviral defense against Cypovirus 1 (Reoviridae) infection in the silkworm, *Bombyx mori. Arch*. Insect Biochem Physiol. (2020) 103:e21616. 10.1002/arch.21616 31502703

[88] Li G Zhou K Zhao G Qian H Xu A. Transcriptome-wide analysis of the difference of alternative splicing in susceptible and resistant silkworm strains after BmNPV infection. 3 Biotech. (2019) 9:152. 10.1007/s13205-019-1669-9 30944799 PMC6434002

[89] Li G Qian H Luo X Xu P Yang J Liu M. Transcriptomic analysis of resistant and susceptible *Bombyx mori* strains following BmNPV infection provides insights into the antiviral mechanisms. Int J Genomics. (2016) 2016:2086346. 10.1155/2016/2086346 27195279 PMC4852350

[90] Guo R Wang S Xue R Cao G Hu X Huang M. The gene expression profile of resistant and susceptible *Bombyx mori* strains reveals cypovirus-associated variations in host gene transcript levels. Appl Microbiol Biotechnol. (2015) 99:5175–87. 10.1007/s00253-015-6634-x 25957492

[91] Zografidis A Van Nieuwerburgh F Kolliopoulou A Apostolou-Karampelis K Head SR Deforce D. Viral Small-RNA analysis of *Bombyx mori* larval midgut during persistent and pathogenic cytoplasmic polyhedrosis virus infection. J Virol. (2015) 89:11473–86. 10.1128/JVI.01695-15 26339065 PMC4645660

[92] Hu ZG Dong ZQ Dong FF Zhu Y Chen P Lu C. Identification of a PP2A gene in *Bombyx mori* with antiviral function against *B. mori* nucleopolyhedrovirus. Insect Sci. (2019) 27:687–96. 10.1111/1744-7917 31070299

[93] Hua X Li B Song L Hu C Li X Wang D. Stimulator of interferon genes (STING) provides insect antiviral immunity by promoting dredd caspase-mediated NF-kappaB activation. J Biol Chem. (2018) 293:11878–90. 10.1074/jbc.RA117.000194 29875158 PMC6066306

[94] Liu TH Dong XL Pan CX Du GY Wu YF Yang JG. A newly discovered member of the Atlastin family, BmAtlastin-n, has an antiviral effect against BmNPV in *Bombyx mori*. Sci Rep. (2016) 6:28946. 10.1038/srep28946 27353084 PMC4926086

[95] Selot R Kumar V Shukla S Chandrakuntal K Brahmaraju M Dandin SB. Identification of a soluble NADPH oxidoreductase (BmNOX) with antiviral activities in the gut juice of *Bombyx mori*. Biosci Biotechnol Biochem. (2007) 71:200–5. 10.1271/bbb.60450 17213661

[96] Ponnuvel KM Nakazawa H Furukawa S Asaoka A Ishibashi J Tanaka H. A lipase isolated from the silkworm *Bombyx mori* shows antiviral activity against nucleopolyhedrovirus. J. Virol. (2003) 77:10725–9. 10.1128/JVI.77.19.10725-10729.2003 12970462 PMC228431

[97] Zhao P Xia F Jiang L Guo H Xu G Sun Q. Enhanced antiviral immunity against *Bombyx mori* cytoplasmic polyhedrosis virus via overexpression of peptidoglycan recognition protein S2 in transgenic silkworms. Dev Comp Immunol. (2018) 87:84–9. 10.1016/j.dci.2018.05.021 29902708

[98] Bao YY Tang XD Lv ZY Wang XY Tian CH Xu YP. Gene expression profiling of resistant and susceptible *Bombyx mori* strains reveals nucleopolyhedrovirus-associated variations in host gene transcript levels. Genomics. (2009) 94:138–45. 10.1016/j.ygeno.2009.04.003 19389468

[99] Kolliopoulou A Van Nieuwerburgh F Stravopodis DJ Deforce D Swevers L Smagghe G. Transcriptome analysis of *Bombyx mori* larval midgut during persistent and pathogenic cytoplasmic polyhedrosis virus infection. PLoS ONE. (2015) 10:e0121447. 10.1371/journal.pone.0121447 25816294 PMC4376736

[100] Bao YY Lv ZY Liu ZB Xue J Xu YP Zhang CX. Comparative analysis of *Bombyx mori* nucleopolyhedrovirus responsive genes in fat body and haemocyte of *B. mori* resistant and susceptible strains. Insect Mol Biol. (2010) 19:347–58. 10.1111/j.1365-2583.2010.00993.x 20201979

[101] Sun Q Guo H Xia Q Jiang L Zhao P. Transcriptome analysis of the immune response of silkworm at the early stage of *Bombyx mori* bidensovirus infection. Dev Comp Immunol. (2020) 106:103601. 10.1016/j.dci.2019.103601 31899306

[102] Xue J Qiao N Zhang W Cheng RL Zhang XQ Bao YY. Dynamic interactions between *Bombyx mori* nucleopolyhedrovirus and its host cells revealed by transcriptome analysis. J Virol. (2012) 86:7345–7359. 10.1128/JVI.07217-12 22532689 PMC3416345

[103] Sagisaka A Fujita K Nakamura Y Ishibashi J Noda H Imanishi S. Genome-wide analysis of host gene expression in the silkworm cells infected with *Bombyx mori* nucleopolyhedrovirus. Virus Res. (2010) 147:166–75. 10.1016/j.virusres.2009.10.015 19883703

[104] Gao K Deng XY Qian HY Qin G Guo XJ. Digital gene expression analysis in the midgut of 4008 silkworm strain infected with cytoplasmic polyhedrosis virus. J. Invertebr Pathol. (2014) 115:8–13. 10.1016/j.jip.2013.10.016 24211674

[105] Jiang L Peng Z Guo Y Cheng T Guo H Sun Q. Transcriptome analysis of interactions between silkworm and cytoplasmic polyhedrosis virus. Sci Rep. (2016) 6:24894. 10.1038/srep24894 27118345 PMC4847007

[106] Feng M Zhang X. Immunity to *Avian leukosis* virus: where are we now and what should we do? Front Immunol. (2016) 7:624. 10.3389/fimmu.2016.00624 28066434 PMC5174080

[107] Wang X Zhang Y Zhang R Zhang J. The diversity of pattern recognition receptors (PRRs) involved with insect defense against pathogens. Curr Opin Insect Sci. (2019) 33:105–10. 10.1016/j.cois.2019.05.004 31358188

[108] Lu Y Su F Li Q Zhang J Li Y Tang T. Pattern recognition receptors in *Drosophila* immune responses. Dev Comp Immunol. (2020) 102:103468. 10.1016/j.dci.2019.103468 31430488

[109] Jiang L Liu W Guo H Dang Y Cheng T Yang W. Distinct functions of *Bombyx mori* peptidoglycan recognition protein 2 in immune responses to bacteria and viruses. Front Immunol. (2019) 10:776. 10.3389/fimmu.2019.00776 31031766 PMC6473039

[110] Lamiable O Imler JL. Induced antiviral innate immunity in *Drosophila*. Curr Opin Microbiol. (2014) 20:62–8. 10.1016/j.mib.2014.05.006 24907422 PMC4133299

[111] Liu W Liu J Lu Y Gong Y Zhu M Chen F. Immune signaling pathways activated in response to different pathogenic micro-organisms in *Bombyx mori*. Mol Immunol. (2015) 65:391–7. 10.1016/j.molimm.2015.02.018 25745806

[112] Wang L Cappelle K Santos D Vanden Broeck J Smagghe G Swevers L. Short-term persistence precedes pathogenic infection: infection kinetics of cricket paralysis virus in silkworm-derived Bm5 cells. J Insect Physiol. (2019) 115:1–11. 10.1016/j.jinsphys.2019.03.004 30905610

